# Beyond the scalpel: humanistic training and professional identity formation in postgraduate plastic surgery education

**DOI:** 10.3389/fmed.2026.1922150

**Published:** 2026-08-25

**Authors:** Zhengbing Guo, Yang Li, Han Chen, Xing Fan

**Affiliations:** 1 Department of Plastic and Reconstructive Surgery, Xijing Hospital, Fourth Military Medical University, Xi’an, Shaanxi, China; 2 School of Humanities and Social Science, Xi'an Jiaotong University, Xi’an, Shaanxi, China

**Keywords:** medical humanities, narrative medicine, plastic surgery education, postgraduate, medical education, professional identity formation

## Abstract

**Objective:**

Postgraduate plastic surgery education has traditionally emphasized anatomical knowledge, operative technique, aesthetic judgment, and procedural competence. However, plastic surgery practice is also shaped by aesthetic expectations, body image concerns, psychosocial vulnerability, medico-legal risk, and commercialized doctor-patient relationships. These tensions suggest that technical training alone is insufficient for preparing trainees to manage the humanistic challenges of contemporary plastic surgery. This study aimed to develop a Professional Identity Formation (PIF)-based humanistic framework for postgraduate plastic surgery education.

**Methods:**

This study adopted a review and framework-development design. First, bibliometric mapping was conducted using records from the Web of Science Core Collection to identify the thematic structure of medical humanities education. Second, literature on litigation, malpractice claims, patient complaints, negative reviews, and medico-legal risks in plastic surgery was synthesized to clarify the specialty-specific conflict structure. Finally, the two evidence streams were integrated through the lens of PIF to construct a humanistic training framework for postgraduate plastic surgery education.

**Results:**

The keyword co-occurrence analysis identified four major thematic clusters in medical humanities education: reflection-driven clinical learning, narrative-based patient understanding, professional identity and sustainable empathy, and contextualized whole-person care. The synthesis of plastic surgery conflict literature showed that disputes are rarely caused by technical failure alone, but are shaped by outcome-expectation gaps, complications, informed-consent and documentation problems, communication breakdowns, commercialization and professional-boundary tensions, and system-process failures. Integrating these findings, this study proposes a PIF-based humanistic framework that connects reflective learning, patient narratives, sustainable empathy, whole-person care, and professional conscience with the real clinical challenges of plastic surgery training.

**Conclusion:**

Humanistic training in plastic surgery should not be treated as an optional supplement to technical education. Rather, it should be understood as a core process through which trainees develop ethical judgment, professional boundaries, emotional resilience, patient-centered responsibility, and a stable professional identity.

## 1 Introduction

Postgraduate plastic surgery education has traditionally been organized around the mastery of anatomical knowledge, operative technique, microsurgical precision, aesthetic judgment, and procedural competence, as reflected in residency milestones, structured surgical exposure, and specialty-specific training requirements (1–3). This technically intensive model has contributed to the standardization and professionalization of plastic surgery training, particularly through case-based learning, structured rotations, competency-oriented assessment, and increasingly formalized curricula in aesthetic surgery and microsurgery (4, 5). However, a fundamental paradox remains: while postgraduate training seeks to produce certainty, reproducibility, and measurable technical competence, real-world plastic surgery practice is often shaped by subjective and unstable factors, including aesthetic preference, body image, psychosocial vulnerability, social comparison, and consumer expectations (6, 7). This paradox is especially visible in aesthetic surgery, where patients’ motivations may be influenced not only by anatomical concerns, but also by social media exposure, cultural beauty ideals, appearance-related distress, and the expectation that bodily modification will produce broader psychosocial transformation (8). As a result, young plastic surgeons may enter clinical practice with strong operative preparation but limited conceptual and educational support for interpreting the ambiguous human meanings behind surgical requests (9). The central challenge of postgraduate plastic surgery education, therefore, is not only how to train technically competent surgeons, but also how to prepare trainees to navigate the uncertain, relational, aesthetic, psychological, and ethical dimensions of plastic surgical care.

This mismatch between technical training and clinical complexity is not merely a theoretical concern; it is repeatedly reflected in medicolegal, complaint-based, and health-system evidence from plastic surgery. National claims data from the NHS suggest that plastic surgery litigation is shaped not only by clinical complications, but also by delays in treatment, communication failures, consent problems, and weaknesses in perioperative care pathways (10). Studies of post-bariatric body-contouring litigation further show that disputes may arise when patients misunderstand the complexity of reconstructive procedures and evaluate postoperative outcomes through the expectations of ordinary aesthetic surgery (11). Patient-facing evidence points in the same direction: negative online reviews of plastic surgeons are frequently driven by nonclinical factors such as bedside manner, staff attitude, billing issues, and perceived service quality, rather than clinical outcomes alone (12). These findings expose a critical educational gap. Communication, ethics, expectation management, psychosocial assessment, and professional boundary-setting are often left to the hidden curriculum of surgical training, even though recent surgical education reviews emphasize that ethics, professionalism, power dynamics, medical errors, and mistreatment require explicit curricular attention and structured educational interventions (13). This gap may intensify plastic surgery’s distinctive “technical-aesthetic-psychological-ethical-relational” conflicts and leave young surgeons underprepared for the emotional, moral, and communicative demands of real-world practice.

Medical humanities offer a promising set of educational resources for addressing this gap, but their value for plastic surgery training cannot be realized through simple transplantation. In broader health professions education, empirical intervention studies have shown that narrative medicine can strengthen learners’ professional identity, self-reflection, emotional expression, and reflective writing capacity by guiding them to attend to, represent, and respond to human suffering (14). Randomized controlled evidence also suggests that narrative medicine teaching and narrative writing can improve professionalism, empathy, and humanistic caring ability, indicating that humanities-based education can be translated into measurable educational outcomes when it is structured as an active learning process rather than a purely theoretical course (15). Other recent intervention studies further show that empathy portfolios may support professional identity formation, while improvisational training can enhance perspective-taking and reduce personal distress in medical students (16). At the same time, longitudinal and technology-enhanced studies indicate that humanities-related education does not produce uniform effects across all learners or settings, and that its impact depends on curriculum design, clinical relevance, learner engagement, and the specific human experience being simulated or interpreted (17). These findings suggest that medical humanities should be understood as a set of adaptable pedagogical resources, rather than as a fixed package of courses that can be directly transferred across specialties. This point is particularly important for postgraduate plastic surgery education. Plastic surgery presents a distinctive educational setting in which clinical judgment is inseparable from aesthetic expectation, body image, commercial influence, risk communication, and professional boundary-setting. Therefore, narrative medicine, reflective practice, empathy training, and professional identity formation should not be imported into plastic surgery as generic modules. They need to be reinterpreted in relation to the specialty’s real educational problems, including how trainees listen to patients’ aesthetic narratives, manage unrealistic expectations, communicate uncertainty, respond to dissatisfaction, and protect professional judgment in commercialized clinical environments.

Building on this logic, this study develops a Professional Identity Formation framework for humanistic training in postgraduate plastic surgery education. PIF provides an appropriate theoretical lens because the central challenge is not merely to teach trainees additional communication techniques or ethical principles, but to support their transformation into plastic surgeons who can integrate technical excellence with patient understanding, professional boundaries, moral responsibility, and emotional resilience. In medical education, PIF emphasizes how learners gradually internalize the values, norms, responsibilities, and ways of being associated with the medical profession through clinical socialization, role modelling, reflection, supervision, and participation in professional communities (18). Applying this perspective to plastic surgery, the present study first identifies four major themes in the medical humanities education literature: narrative medicine and patient illness experience, reflective practice and core competency development, professional identity formation and emotional resilience, and clinical contextualization with whole-person care. It then relates these themes to the distinctive conflict structure of plastic surgery, including aesthetic expectation gaps, risk communication, informed consent, psychosocial vulnerability, commercialization, and unstable doctor-patient relationships. Through this integration, the study aims to construct a specialty-adapted humanistic training framework that helps postgraduate plastic surgery trainees move from being technically competent operators toward becoming reflective, ethically grounded, patient-centered, and professionally mature plastic surgeons.

## 2 Methods

### 2.1 Data sources

The Web of Science Core Collection was selected as the primary database for this study. This database provides standardized bibliographic records, structured citation information, author keywords, Keywords Plus, publication metadata, and export formats suitable for keyword co-occurrence analysis and literature mapping. Because the first stage of this study aimed to identify the thematic structure of medical humanities education, a database with consistent indexing and reliable metadata was necessary. In addition, Web of Science covers a wide range of journals in medical education, health professions education, clinical medicine, surgery, ethics, and health services research, making it appropriate for capturing both the broader medical humanities education literature and the specialty-specific literature on plastic surgery conflicts.

This study drew on two bodies of literature. The first dataset was used to map the broader field of medical humanities education. The search focused on three established educational approaches that are particularly relevant to postgraduate clinical training: narrative medicine, Balint-based education, and reflective practice. These three approaches were selected because they represent complementary pathways through which medical humanities have been translated into clinical education. Narrative medicine emphasizes patients’ illness experiences, meanings, and stories, and is therefore closely related to patient understanding, empathy, and communication. Balint-based education focuses on doctor-patient relationships, emotional reflection, and the interpersonal dimensions of clinical encounters. Reflective practice emphasizes the transformation of clinical experience into professional learning, ethical awareness, and clinical judgment. Together, these three strands provide a focused yet sufficiently broad entry point for identifying humanistic educational resources relevant to postgraduate medical training. Other related concepts, such as empathy, communication, professionalism, patient experience, clinical reasoning, and professional identity formation, were incorporated during screening, keyword analysis, and thematic interpretation rather than being treated as separate initial search blocks.

The search strategy for the medical humanities education dataset included the following three search blocks: TS = (“Narrative Medicine”); TS = (“Balint Group*” OR “Balint Seminar*” OR “Balint Work*”); and TS = (“Reflective Practice” OR “Reflective Learning” OR “Reflective Journal*”) AND TS = (“Medical Education” OR “Graduate Medical Education” OR “Residency” OR “Clinical Teaching”). The full search strategy is presented in Table 1. The initial search yielded 1,421 records.

**Table 1:** Search strategy and screening results.

Search component	Search strategy	Initial records	Final records used
Medical humanities education	TS = (“Narrative Medicine”) OR TS = (“Balint Group*” OR “Balint Seminar*” OR “Balint Work*”) OR (TS = (“Reflective Practice” OR “Reflective Learning” OR “Reflective Journal*”) AND TS = (“Medical Education” OR “Graduate Medical Education” OR “Residency” OR “Clinical Teaching”))	1,421	1,003
Plastic surgery conflicts	TS = (“Plastic Surgery” OR “Cosmetic Surgery” OR “Aesthetic Surgery” OR “Reconstructive Surgery”) AND TS = (“Dispute*” OR “Doctor-Patient” OR “compensation*”)	186	13

The second dataset was used to identify the distinctive conflict structure of plastic surgery practice. A targeted search was conducted in the Web of Science Core Collection to retrieve studies on doctor-patient disputes, medico-legal claims, litigation, patient complaints, compensation, and related conflict issues in plastic surgery. The search focused on plastic surgery, cosmetic surgery, aesthetic surgery, and reconstructive surgery in relation to disputes, doctor-patient relationships, and compensation. The search strategy was: TS = (“Plastic Surgery” OR “Cosmetic Surgery” OR “Aesthetic Surgery” OR “Reconstructive Surgery”) AND TS = (“Dispute*” OR “Doctor-Patient” OR “compensation*”). The detailed search strategy is also provided in Table 1. The initial search yielded 186 records.

### 2.2 Screening process and study selection

**Figure 1:**
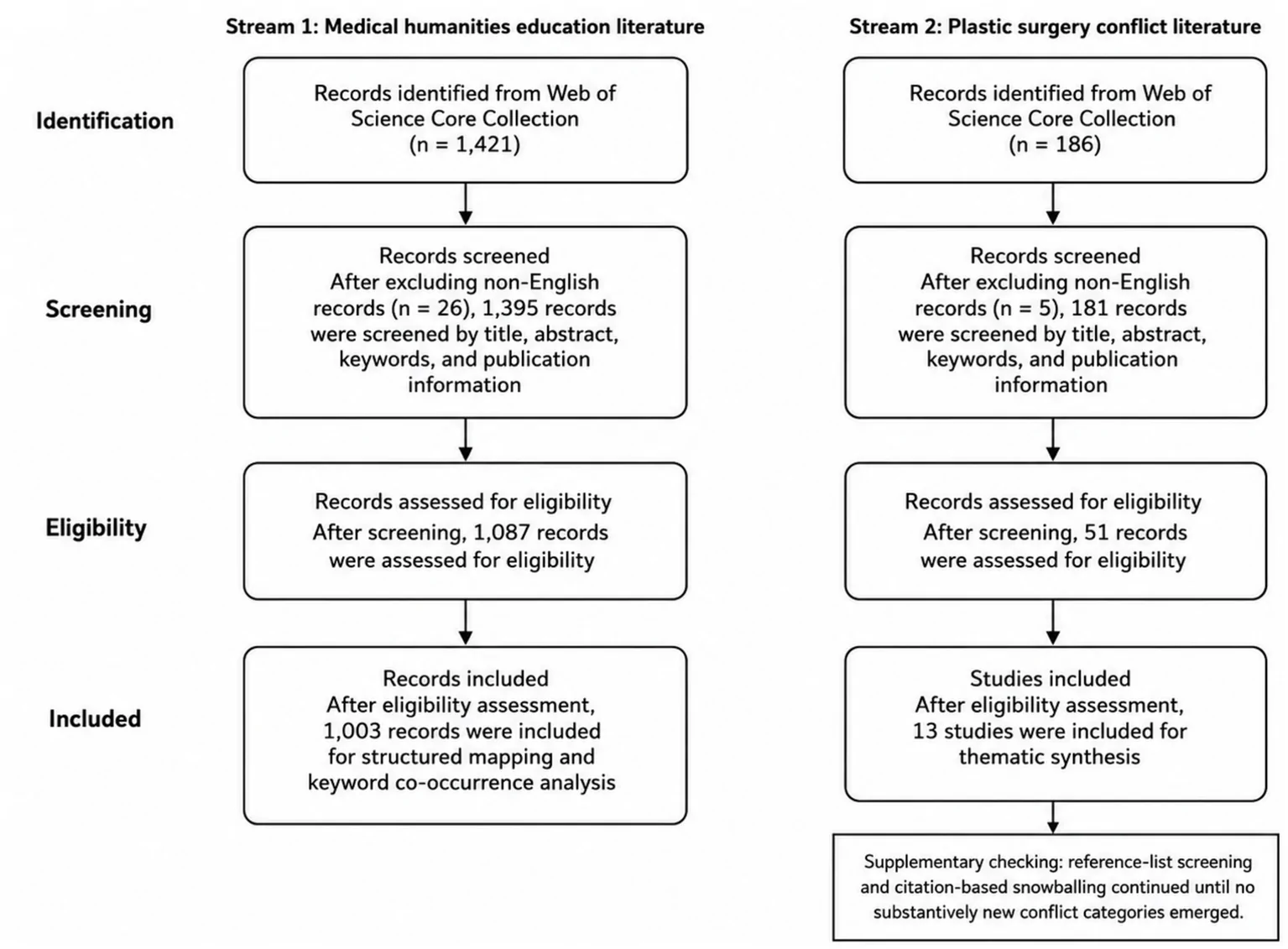
Literature identification and screening process.

The literature identification and screening process followed a PRISMA-style structure, including identification, screening, eligibility assessment, and inclusion. Because this study was designed as a structured review and framework-development study rather than a full systematic review or meta-analysis, the flow diagram was used to improve transparency in documenting the two evidence streams rather than to claim full PRISMA compliance. The screening process is summarized in Figure 1.

For the medical humanities education stream, 1,421 records were identified from the Web of Science Core Collection. Before screening, 26 non-English records were removed. The remaining 1,395 records were screened by title, abstract, keywords, and publication information, and 308 records were excluded because they were clearly outside the scope of medical humanities education, clinical teaching, postgraduate medical education, or health professions education. A total of 1,087 records were then assessed for eligibility, of which 84 records were excluded because they were not sufficiently relevant to medical humanities education, postgraduate clinical training, or humanistic approaches in health professions education. Finally, 1,003 records were included for structured literature mapping and keyword co-occurrence analysis. Eligible records included studies on narrative medicine, reflective practice, reflective writing, Balint groups, empathy, communication, professionalism, patient experience, clinical reasoning, professional identity formation, and other humanistic approaches to medical education, graduate medical education, residency training, or clinical teaching.

For the plastic surgery conflict stream, 186 records were identified from the Web of Science Core Collection. Before screening, 5 non-English records were removed. The remaining 181 records were screened by title, abstract, keywords, and publication information, and 130 records were excluded because they were clearly outside the scope of plastic surgery conflicts, doctor-patient disputes, complaints, litigation, compensation, informed consent, communication, or medico-legal risk. A total of 51 records were then assessed for eligibility, of which 38 records were excluded because they did not provide sufficient relevance to the thematic synthesis of plastic surgery conflicts. Finally, 13 studies were included for thematic synthesis. Studies were retained if they addressed malpractice claims, judicial decisions, patient complaints, negative patient reviews, informed consent, communication breakdowns, expectation-related dissatisfaction, postoperative care problems, compensation, medico-legal risk, or commercialized doctor-patient relationships in plastic surgery. Because the literature on plastic surgery conflicts is scattered across surgical journals, legal medicine, health services research, and specialty-specific outlets, reference-list screening and citation-based snowballing were used as supplementary checking procedures to determine whether additional relevant studies or substantively new conflict types could be identified. Snowballing continued until no new recurring conflict categories emerged.

### 2.3 Analysis strategy

This study adopted a structured review and framework-development design. The analysis was conducted in three sequential but interrelated stages. In the first stage, the medical humanities education dataset was analyzed through structured literature mapping and keyword co-occurrence analysis. Author keywords and Keywords Plus were extracted from the bibliographic records to identify recurring conceptual patterns within the broader field of medical humanities education. The resulting keyword clusters were then interpreted thematically in relation to their educational meanings. This process generated four major thematic domains: reflection-driven clinical learning, narrative-based patient understanding, professional identity and sustainable empathy, and contextualized whole-person care. These domains were treated as transferable pedagogical resources rather than as ready-made curricular modules.

In the second stage, the plastic surgery conflict literature was analyzed through thematic synthesis. For each included study, information was extracted on the clinical context, data source, type of dispute or complaint, conflict triggers, medico-legal factors, informed-consent or documentation issues, communication problems, commercial or professional-boundary concerns, and implications for education. These extracted items were first coded descriptively and then compared across studies to identify recurring conflict patterns. Through this process, six major conflict themes were developed: outcome-expectation conflict, complication-related conflict, informed-consent and documentation conflict, communication and relationship conflict, commercialization and professional-boundary conflict, and system-process conflict. These themes were understood as overlapping dimensions of conflict in plastic surgery practice rather than as mutually exclusive categories.

**Figure 2:**
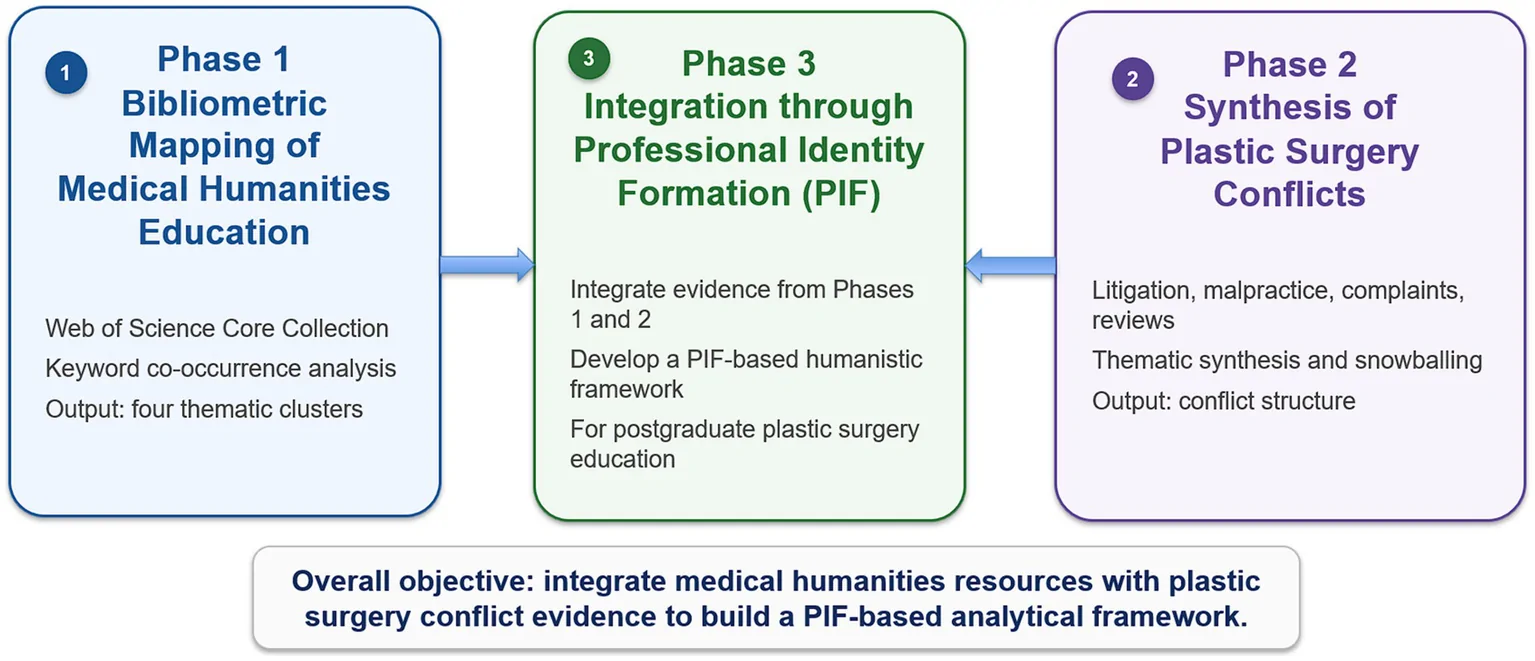
Analytical strategy of the study.

In the third stage, the two evidence streams were integrated through the lens of Professional Identity Formation (PIF). In this study, PIF functioned first as the theoretical framework for understanding humanistic training as a process of physician formation rather than as the acquisition of isolated communication or ethical skills. It then functioned as an analytical lens for connecting the four medical humanities themes with the six plastic surgery conflict themes. Specifically, the analysis asked how reflection-driven clinical learning, narrative-based patient understanding, professional identity and sustainable empathy, and contextualized whole-person care could respond to recurrent conflicts in plastic surgery, including expectation gaps, informed-consent and documentation problems, communication breakdowns, commercialization, and professional-boundary tensions. Finally, PIF was treated as the intended educational outcome of the proposed framework, namely the development of plastic surgery trainees who can integrate technical competence with ethical judgment, emotional resilience, patient-centered responsibility, professional conscience, and stable professional boundaries. Through this integrative process, the final PIF-based humanistic framework for postgraduate plastic surgery education was developed, as shown in Figure 2.

## 3 Results

**Figure 3:**
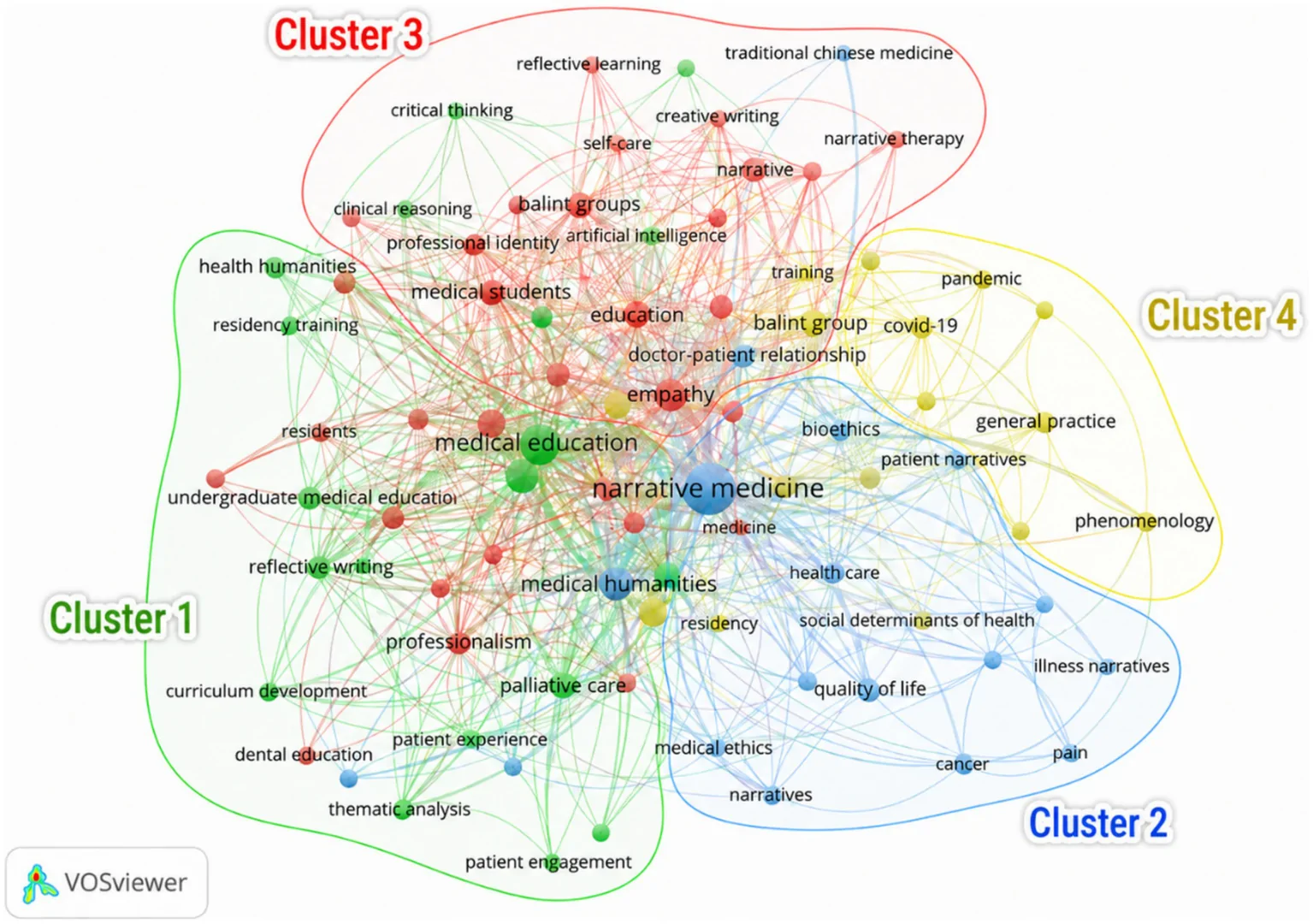
Keyword co-occurrence network of medical humanities education.

The keyword co-occurrence analysis reveals a coherent thematic structure within the field of medical humanities education. Rather than appearing as a loose collection of abstract humanistic concepts, the network is organized around four interrelated clusters, as shown in Figure 3: Cluster 1, the green cluster, labelled “Reflection-Driven Clinical Learning”; Cluster 2, the blue cluster, labelled “Narrative-Based Patient Understanding”; Cluster 3, the red cluster, labelled “Professional Identity and Sustainable Empathy”; and Cluster 4, the yellow cluster, labelled “Contextualized Whole-Person Care.” As shown in Table 2, representative keywords in each cluster further illustrate the conceptual focus of these themes. Together, Figure 3 and Table 2 indicate that medical humanities education has developed into an organized body of pedagogical resources that links how trainees learn, how they understand patients, how they become physicians, and how humanistic capacities are enacted in real clinical contexts. Specifically, the green cluster emphasizes the transformation of clinical experience into reflective learning and professional judgment; the blue cluster highlights the interpretation of patients’ narratives and lived meanings of illness; the red cluster focuses on trainees’ professional identity, empathy, resilience, and emotional sustainability; and the yellow cluster shows how humanistic capacities are translated into person-centered and relationship-sensitive clinical care. For the purpose of this study, these four clusters provide the conceptual foundation for examining how broader medical humanities resources can be adapted to postgraduate plastic surgery education.

**Table 2:** Representative keywords by cluster.

Cluster 1 Green	Cluster 2 Blue	Cluster 3 Red	Cluster 4 Yellow
Medical education	Narrative medicine	Empathy	General practice
Reflective practice	Medical humanities	Professional identity	Psychiatry
Reflective writing	Narratives	Professional identity formation	Chronic pain
Clinical reasoning	Illness narratives	Professionalism	Phenomenology
Critical thinking	Patient narratives	Medical students	Personalized medicine
Communication	Quality of life	Residents	Well-being
Curriculum development	Medical ethics	Balint groups	Training
Residency training	Doctor-patient relationship	Self-care	COVID-19
Patient experience	Health care	Reflective learning	Pandemic
Patient engagement	Social determinants of health	Narrative therapy	Balint group

### 3.1 Reflection-driven clinical learning

Cluster 1 reveals a shift in medical education from passive clinical exposure to reflection-driven clinical learning, highlighting reflection as a mechanism through which learners transform clinical experience into professional understanding rather than merely accumulating time in clinical settings. The central concern of this cluster is not simply whether learners encounter enough cases, but whether they can interpret those encounters in ways that strengthen self-awareness, ethical sensitivity, patient-centeredness, and clinical judgment. Reflective writing and learning diaries show that students’ reflections often move beyond technical learning to include physician-patient relationships, uncertainty, emotional responses, and the social meaning of care (19). Reflection is also closely connected with professional identity formation, as students’ reflective expressions reveal how they understand themselves as future physicians, process emotions during clinical rotations, and negotiate communication challenges, hierarchy, and cultural complexity in clinical environments (20). This cluster also connects reflection with higher-order thinking, since empirical evidence suggests that reflective capacity is associated with critical thinking disposition and more analytical approaches to clinical problems (21). In parallel, studies of clinical reasoning education indicate that complex judgment improves when learners are given structured opportunities for deliberate practice, feedback, and longitudinal learning rather than relying on clinical exposure alone. Portfolio-based interventions further suggest that reflection, empathy, and identity development can be brought into assessable educational practices when learners are asked to document, revisit, and interpret their own professional growth (16). Taken together, Cluster 1 suggests that reflection should be understood not as a vague humanistic ideal, but as a practical educational mechanism linking experience, reasoning, emotion, feedback, and professional development (22). For postgraduate plastic surgery education, this theme suggests that difficult consultations, expectation management, complications, informed consent, aesthetic judgment, and ethical boundary-setting can be transformed into structured opportunities for clinical learning and professional growth.

### 3.2 Narrative-based patient understanding

Cluster 2 centers on the role of patient narratives in transforming clinical encounters from encounters with diseases into encounters with persons. Its core meaning is that medical humanities education does not simply add stories to biomedical training, but offers a way of understanding how patients give meaning to illness, treatment, vulnerability, and recovery. Through narrative-based learning, students are encouraged to move beyond diagnostic labels and procedural thinking, and to attend to how patients describe suffering, uncertainty, fear, family roles, stigma, hope, and the social consequences of illness (14). This emphasis is important because clinical decisions are not made in a purely biomedical space; they are also shaped by how patients understand their bodies, interpret risks, communicate expectations, and evaluate whether care has responded to what matters in their lives (23). In this sense, narrative competence is not a soft or decorative element of medical education, but a practical capacity for translating subjective experience into clinically meaningful understanding. Existing educational interventions suggest that narrative medicine can strengthen empathy and help learners engage more attentively with patients’ perspectives, while narrative-based teaching can make uncertainty, counseling, and difficult communication more concrete and teachable in clinical education (24, 25). Narrative curricula also require attention to patient agency, because patients’ life stories should not be reduced to educational objects detached from voice, ownership, and consent (26). For postgraduate plastic surgery education, this implication should be applied cautiously but meaningfully. Surgical requests may appear to be anatomical or aesthetic, yet they may also express concerns about body image, social confidence, quality of life, stigma, and the hope for bodily transformation. Patient-reported outcome research in body contouring surgery similarly shows that appearance-related care must be evaluated through patients’ own perceptions of quality of life and satisfaction, not only through technical correction (27). Therefore, this cluster provides a basis for introducing narrative-based patient understanding into plastic surgery training, helping trainees listen more carefully, clarify expectations more responsibly, and preserve clinical judgment while recognizing the person behind the procedure.

### 3.3 Professional identity and sustainable empathy

Cluster 3 shifts the focus from what trainees learn to who they become. Its central concern is professional identity formation, namely the process through which medical learners gradually internalize the values, responsibilities, ethical commitments, and ways of being that define the medical profession. This theme deepens the educational meaning of medical humanities: empathy, professionalism, resilience, and well-being are not isolated soft skills, but interrelated elements of a developing professional self. In this sense, humanistic education is not only designed to improve communication performance; it also supports the formation of physicians who can sustain moral judgment, patient-centered responsibility, and professional boundaries under pressure. This view is consistent with the broader PIF literature, which emphasizes that professional identity develops through clinical socialization, role modelling, reflection, mentoring, communities of practice, and emotionally meaningful encounters with patients and colleagues (28–30). It also aligns with work on humanistic and resilient physicians, which argues that learners need educational spaces to integrate professional values with emotional experience rather than simply perform professionalism as external behavior (31). The cluster therefore suggests that empathy should be understood as a professional capacity that must be cultivated and protected, not as an unlimited emotional resource. In clinical training, repeated exposure to suffering, uncertainty, conflict, workload, and hierarchical pressure may reshape how learners relate to patients and to themselves; without support, empathy may become depleted or defensive rather than sustainable (32). For postgraduate plastic surgery education, this theme is especially relevant because trainees work in a field where technical and aesthetic performance is entangled with patient vulnerability, appearance-related distress, unrealistic expectations, commercial pressures, and fear of complaints. Existing evidence suggests that ethics and professionalism training in plastic surgery residency remains uneven, while burnout and emotional exhaustion are real concerns among plastic surgery trainees and surgeons (33). Professional identity therefore functions as a stabilizing anchor: it helps trainees understand themselves not as technical service providers responding to consumer demand, but as healthcare professionals responsible for patient protection, ethical judgment, and long-term well-being. Sustainable empathy in plastic surgery does not mean satisfying every aesthetic request or absorbing unlimited emotional labor; it means recognizing patients’ vulnerability while preserving professional boundaries, emotional resilience, and moral responsibility.

### 3.4 Contextualized whole-person care

Cluster 4 shifts medical humanities from educational ideals to the lived clinical contexts in which care is actually delivered. Its central concern is that humanistic competence should not remain at the level of classroom reflection, communication exercises, or abstract ethical discussion, but should become part of everyday clinical judgment. In this cluster, care is no longer understood only as diagnosing a disease or performing an intervention; it is framed as a contextual practice in which clinicians must consider bodily symptoms, emotional suffering, social relationships, life goals, values, vulnerability, and the patient’s own understanding of what recovery means. This orientation resonates with recent work on person-centered care in health professions education, which treats person-centredness as a teachable clinical and educational responsibility rather than a general moral slogan (34). For postgraduate plastic surgery education, this theme is especially relevant because surgical requests often sit at the boundary between anatomical concern, aesthetic desire, body image, psychosocial adaptation, and consumer-oriented expectation. A contextualized whole-person approach helps trainees ask not only whether a procedure is technically possible, but whether it is clinically appropriate, psychologically safe, ethically justified, and aligned with the patient’s long-term well-being. This is particularly important in aesthetic and reconstructive practice, where body dysmorphic concerns, appearance-related distress, and unrealistic expectations may complicate patient selection, decision-making, and postoperative satisfaction (35). At the same time, patient-reported outcome research reminds us that plastic surgery outcomes cannot be evaluated solely through technical correction, because patients’ own perceptions of appearance, quality of life, and satisfaction are central to the meaning of successful care (27). Thus, Cluster 4 supports a clinical-humanistic training orientation that helps trainees evaluate the whole person behind the surgical request, combining psychosocial screening, risk communication, ethical judgment, and referral awareness without reducing care either to technical repair or consumer satisfaction.

### 3.5 From surgical conflicts to humanistic training needs

The conflict literature suggests that plastic surgery disputes should be understood as multi-layered events rather than as direct consequences of technical failure. As summarized in Table 3, these conflicts can be grouped into several recurring types: outcome-expectation conflict, complication-related conflict, informed-consent and documentation conflict, communication and relationship conflict, commercialization and professional-boundary conflict, and system-process conflict. Across these types, disputes frequently emerge from the interaction of surgical risk, aesthetic expectation, informed consent, documentation, communication quality, postoperative follow-up, and the commercialized context of aesthetic medicine. Studies on aesthetic body surgery litigation, plastic surgery disputes in China, malpractice claims, and judicial decisions show that dissatisfaction with surgical outcomes, postoperative complications, deficient consent processes, weak medical records, and ineffective doctor-patient communication are repeatedly associated with disputes, compensation, or unfavorable legal outcomes (36–39). These findings indicate that conflict in plastic surgery is often shaped by outcome-expectation gaps: a technically acceptable result may still be interpreted as failure if the patient’s desired body image, aesthetic expectations, and understanding of risk have not been adequately explored, negotiated, and documented.

**Table 3:** Major conflict types in plastic surgery and their educational implications.

Conflict type	Typical triggers	Representative sources
Outcome-expectation conflict	Dissatisfaction with aesthetic results; mismatch between expected and actual appearance; perceived failure despite technically acceptable outcomes	Mussabekova et al. (36); Su et al. (37); Vila-Nova da Silva et al. (39)
Complication-related conflict	Postoperative complications; revision surgery; scarring; functional problems; perceived negligence after adverse outcomes	Remington et al. (38); Mussabekova et al. (36); Daneshi et al. (10)
Informed-consent and documentation conflict	Inadequate consent; insufficient preoperative assessment; weak medical records; failure to document risks and alternatives	Su et al. (37); Remington et al. (38); Vila-Nova da Silva et al. (39)
Communication and relationship conflict	Poor doctor-patient communication; staff attitude; perceived lack of care; insufficient explanation; weak follow-up	Patel and Morrison (52); Ding et al. (40); Qin et al. (53); Prasad et al. (12)
Commercialization and professional-boundary conflict	Provider-consumer framing; advertising issues; pressure to satisfy patient demand; service satisfaction replacing medical judgment	Mariani et al., (41); Prasad et al. (12); Facchin et al. (11)
System-process and care-pathway conflict	Delays in treatment; billing problems; poor coordination; weak care pathways; system-level failures	Daneshi et al. (10); Taniguchi, et al. (42); Patel and Morrison (52)

A second layer of conflict concerns the relational, institutional, and professional environment in which plastic surgery care is delivered. Evidence from complaint studies, laser treatment centers, emergency trauma suturing, online review platforms, medical-board complaints, post-bariatric body-contouring litigation, NHS claims, and Japanese malpractice trends suggests that patients often object not only to clinical outcomes, but also to staff attitude, billing, waiting time, communication style, perceived lack of care, treatment delays, insufficient follow-up, advertising practices, professional conduct, care pathways, and system-level failures (40–42). In aesthetic practice, these tensions may be intensified by commercialization, as the doctor-patient relationship can be reframed as a provider-consumer transaction and surgeons may be judged through service satisfaction rather than medical judgment alone. Taken together, this body of literature provides a concrete clinical rationale for integrating narrative medicine, reflective practice, patient-centered care, and professional identity formation into postgraduate plastic surgery education. Humanistic training is therefore not an optional supplement to technical instruction, but a necessary educational response to the real conflict structure of plastic surgery practice.

## 4 Discussion

In this study, PIF serves three interrelated functions. First, it provides the theoretical framework for understanding why humanistic education in plastic surgery should not be limited to communication skills, empathy training, or ethical instruction alone. The central concern is not only whether trainees can perform specific professional behaviors, but whether they gradually internalize the values, responsibilities, moral commitments, emotional dispositions, and professional boundaries required of plastic surgeons (43). Second, PIF serves as an analytical lens that links the broader medical humanities education literature with the specialty-specific conflict structure of plastic surgery. The four medical humanities themes identify educational resources for reflection, patient understanding, sustainable empathy, and whole-person care, while the six conflict themes identify the clinical and medico-legal situations in which these resources are most needed (44). Third, PIF represents the intended educational outcome of the proposed framework: the formation of plastic surgery trainees who can combine operative competence with ethical judgment, professional conscience, emotional resilience, and patient-centered responsibility without reducing care to consumer satisfaction. In this sense, PIF is not an additional topic placed beside medical humanities and plastic surgery conflicts, but the integrative mechanism through which these two bodies of evidence are connected.

### 4.1 PIF-oriented humanistic training framework

**Figure 4:**
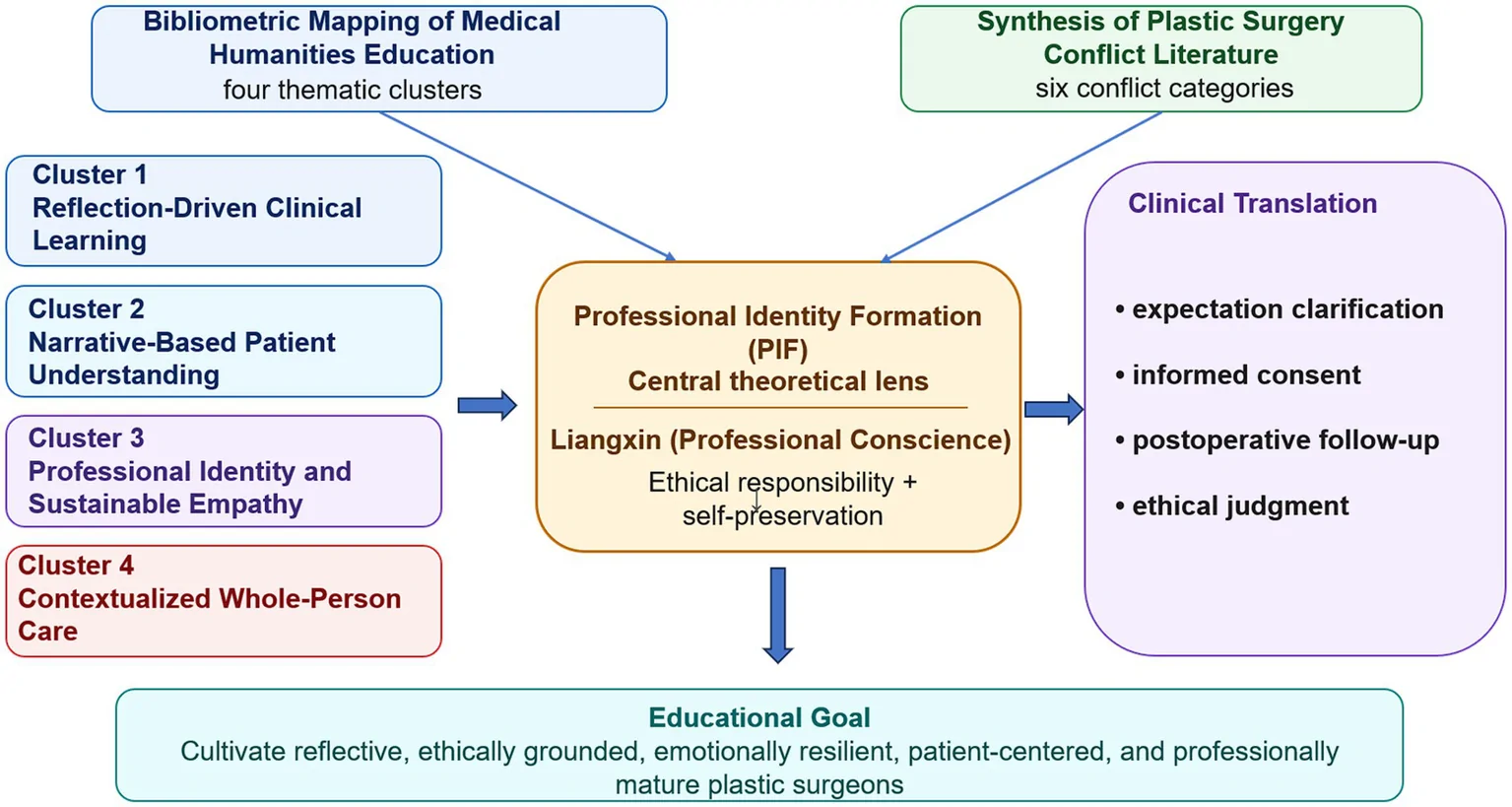
PIF-Based framework for plastic surgery education.

Building on the four keyword clusters and the synthesis of plastic surgery conflicts, this study proposes a PIF-based humanistic framework for postgraduate plastic surgery education, as shown in Figure 4. The bibliometric analysis shows that medical humanities education is organized around four transferable pedagogical resources: reflection-driven clinical learning, narrative-based patient understanding, professional identity and sustainable empathy, and contextualized whole-person care. The conflict synthesis further shows that plastic surgery disputes are not caused by technical failure alone, but are produced through outcome-expectation gaps, complications, informed-consent and documentation problems, communication breakdowns, commercialization and professional-boundary tensions, and system-process failures. These findings suggest that the humanistic challenges of plastic surgery cannot be addressed by adding isolated communication skills to a technical curriculum. Instead, they require a framework that connects how trainees learn from clinical experience, how they understand patients, how they form a professional self, and how they translate these capacities into real clinical judgment.

Professional Identity Formation provides the central theoretical lens for this framework because the key educational question is not only what postgraduate trainees can do, but what kind of plastic surgeons they are becoming. PIF emphasizes the process through which learners internalize professional values, ethical commitments, responsibilities, emotional dispositions, and ways of being associated with the medical profession (18). Compared with competency-based medical education, which primarily focuses on observable competencies, milestones, and task performance, PIF more directly addresses the formation of professional values, moral judgment, emotional resilience, and professional boundaries (45). This distinction is especially important in plastic surgery, where trainees must balance technical feasibility with aesthetic expectation, patient autonomy with professional responsibility, and service responsiveness with ethical boundary-setting. In the Chinese medical education context, this framework can be further enriched by the concept of Liangxin, understood here as professional conscience. Liangxin refers to an internal moral orientation that enables trainees to act responsibly when external supervision is absent, protect patients’ long-term well-being when commercial incentives are present, and preserve ethical boundaries when patient demands are strong (46, 47). Importantly, Liangxin should not be interpreted as endless self-sacrifice or unlimited emotional labor. Rather, it links responsibility with self-preservation: humanistic training should help trainees remain humane without becoming self-erasing, patient-centered without becoming consumer-driven, and ethically firm without becoming emotionally detached.

As shown in Figure 4, the proposed framework integrates four mutually reinforcing dimensions. First, reflection-driven clinical learning enables trainees to transform difficult consultations, complications, informed-consent problems, expectation management, and ethical uncertainty into structured professional learning. Second, narrative-based patient understanding helps trainees recognize the stories behind surgical requests, including body image concerns, stigma, trauma, social confidence, quality-of-life goals, and expectations for bodily transformation. Third, professional identity and sustainable empathy support trainees in maintaining compassion, emotional resilience, and professional boundaries in a specialty marked by high expectations, dissatisfaction, commercial pressure, and medico-legal vulnerability. Fourth, contextualized whole-person care translates these capacities into clinical action through psychosocial screening, risk communication, shared decision-making, expectation clarification, postoperative follow-up, and referral awareness. Taken together, the framework reframes humanistic training as a core process of becoming a plastic surgeon, rather than as an optional moral supplement to technical training. It offers a specialty-adapted pathway for helping postgraduate trainees move from technically competent operators toward reflective, ethically grounded, emotionally resilient, patient-centered, and professionally mature plastic surgeons.

### 4.2 AI as a future support for PIF-based humanistic training

Although AI was not one of the primary evidence streams analyzed in this study, it is important to discuss its future role because both clinical practice and medical education are increasingly embracing AI. In plastic surgery, AI is already being explored for patient communication, image generation, surgical education, documentation, consultation support, and outcome visualization. Therefore, excluding AI entirely would overlook an important emerging context in which future humanistic training is likely to occur. Within the proposed PIF-based framework, however, AI should not be understood as an independent solution or as a replacement for humanistic care. Rather, it may serve as a future educational support for helping trainees rehearse difficult conversations, clarify patient expectations, prepare informed-consent communication, visualize possible outcomes, and reflect on communication risks in a structured way (48–50).

This future implication is particularly relevant to plastic surgery, where clinical encounters often involve aesthetic expectations, body image concerns, postoperative dissatisfaction, commercial pressures, and medico-legal anxiety. For example, AI-based simulation could be used to help trainees practice consultations involving unrealistic expectations, refusal of inappropriate surgical requests, or explanation of scarring and revision risks. However, AI may also reinforce consumer-driven practice if it is used mainly to increase efficiency, satisfy patient demand, or standardize complex patient vulnerability into scripted responses. Therefore, AI should be incorporated into humanistic training only under the guidance of Professional Identity Formation. The moral meaning of care still depends on the surgeon’s reflective judgment, ethical responsibility, professional conscience, emotional presence, and capacity to build therapeutic trust (51).

### 4.3 Practical implications

In practical terms, the proposed framework suggests that humanistic training may be embedded into existing postgraduate plastic surgery teaching activities rather than added as a separate and burdensome course. For example, brief reflective prompts could be incorporated into case discussions, complication reviews, informed consent teaching, postoperative dissatisfaction debriefings, and morbidity and mortality meetings. These prompts may guide trainees to consider whether patient expectations were clarified, risks were communicated effectively, documentation reflected patient understanding, and professional boundaries were maintained.

The framework may also inform preoperative consultation training, simulation-based teaching, faculty feedback, reflective portfolios, and communication training for difficult encounters. Particular attention could be given to high-risk scenarios such as unrealistic aesthetic expectations, scarring and revision risks, inappropriate surgical requests, body image distress, and postoperative dissatisfaction. These suggestions should be understood as educational implications derived from the proposed framework rather than as evidence-tested interventions. Future studies are needed to evaluate their feasibility, acceptability, and impact in real postgraduate plastic surgery training settings.

### 4.4 Future research

Future research can proceed in three directions. First, the proposed PIF-oriented framework should be empirically tested in postgraduate plastic surgery training programs through interviews, Delphi studies, curriculum pilots, and mixed-methods evaluation, with particular attention to whether narrative medicine, reflective practice, and expectation-management training improve trainees’ communication, informed consent, ethical reasoning, and professional identity formation. Second, future studies should develop and validate practical assessment tools for humanistic competence in plastic surgery, including narrative competence, reflective capacity, psychosocial screening, risk communication, management of unrealistic aesthetic expectations, and professional boundary-setting in commercialized clinical contexts. Third, future research should further examine the cultural and technological extension of this framework, especially how the concept of Liangxin can enrich professional conscience in Chinese medical education and how AI-based tools can support consultation simulation, patient education, documentation, and outcome tracking without replacing human judgment, therapeutic trust, and ethical responsibility.

### 4.5 Limitations

This study has several limitations. First, the literature search was conducted primarily in the Web of Science Core Collection. Although this database provides standardized bibliographic records and is suitable for keyword co-occurrence analysis, relevant studies indexed in other databases, such as PubMed, Scopus, Embase, or regional databases, may have been missed. Second, only 13 plastic surgery conflict studies were ultimately included for thematic synthesis. Although supplementary reference-list screening and citation-based snowballing were conducted, the included studies may not fully capture all forms of conflict across different countries, healthcare systems, legal environments, and training contexts.

Third, both the keyword co-occurrence analysis and the thematic synthesis involved interpretive judgment. The identification and naming of the four medical humanities themes and six plastic surgery conflict themes were based on patterns observed in the literature, but they should not be regarded as fixed or exhaustive categories. Fourth, the proposed PIF-based humanistic framework remains conceptual. It represents an interpretive integration of medical humanities education literature and plastic surgery conflict literature, rather than an empirically validated curriculum or intervention. Future studies should test the feasibility, acceptability, and educational impact of this framework in real postgraduate plastic surgery training settings through curriculum pilots, qualitative interviews, longitudinal evaluation, and mixed-methods research.

## 5 Conclusion

This study shows that medical humanities can provide a meaningful educational foundation for postgraduate plastic surgery training, but only when adapted to the specialty’s clinical realities. By integrating reflection-driven clinical learning, narrative-based patient understanding, professional identity and sustainable empathy, and contextualized whole-person care with the conflict structure of plastic surgery, this study proposes a PIF-based humanistic framework. The framework suggests that humanistic training should not be an optional addition to technical education, but a core process for cultivating plastic surgeons who can combine operative competence with ethical judgment, professional conscience, emotional resilience, and patient-centered responsibility.
